# Iron specificity of a biosensor based on fluorescent pyoverdin immobilized in sol-gel glass

**DOI:** 10.1186/1754-1611-5-4

**Published:** 2011-05-10

**Authors:** Michael F Yoder, William S Kisaalita

**Affiliations:** 1 Department of Biological and Agricultural Engineering, Driftmier Engineering Center, University of Georgia Athens GA 30602, USA; 2 William S. Kisaalita, Cellular Bioengineering Laboratory, Faculty of Engineering, University of Georgia Athens GA 30602, USA

**Keywords:** Pyoverdin, fluorescence, siderophore, Pseudomonas aeruginosa, iron, ferrous, ferric, biosensor, sol-gel glass, immobilized

## Abstract

Two current technologies used in biosensor development are very promising: 1. The sol-gel process of making microporous glass at room temperature, and 2. Using a fluorescent compound that undergoes fluorescence quenching in response to a specific analyte. These technologies have been combined to produce an iron biosensor. To optimize the iron (II or III) specificity of an iron biosensor, pyoverdin (a fluorescent siderophore produced by *Pseudomonas *spp.) was immobilized in 3 formulations of porous sol-gel glass. The formulations, A, B, and C, varied in the amount of water added, resulting in respective R values (molar ratio of water:silicon) of 5.6, 8.2, and 10.8. Pyoverdin-doped sol-gel pellets were placed in a flow cell in a fluorometer and the fluorescence quenching was measured as pellets were exposed to 0.28 - 0.56 mM iron (II or III). After 10 minutes of exposure to iron, ferrous ion caused a small fluorescence quenching (89 - 97% of the initial fluorescence, over the range of iron tested) while ferric ion caused much greater quenching (65 - 88%). The most specific and linear response was observed for pyoverdin immobilized in sol-gel C. In contrast, a solution of pyoverdin (3.0 μM) exposed to iron (II or III) for 10 minutes showed an increase in fluorescence (101 - 114%) at low ferrous concentrations (0.45 - 2.18 μM) while exposure to all ferric ion concentrations (0.45 - 3.03 μM) caused quenching. In summary, the iron specificity of pyoverdin was improved by immobilizing it in sol-gel glass C.

## Background

Many medical diagnoses, research studies, and industrial processes could benefit from an economical, rapid, sensitive iron biosensor because of the widespread importance of iron, even at ppb levels. Current techniques for measuring iron may be very accurate [1] but can be very expensive, may involve large pieces of equipment, and the procedures can be time-consuming. Colorimetric methods (using ferrozine or 1,10-phenanthroline) with a spectrophotometer are only specific for ferrous iron, and are generally less accurate than methods such as atomic absorption and plasma emission spectroscopy (which measure total iron). Ion-selective electrodes for iron are not commercially available.

Various metal binding ligands and techniques have been proposed for the determination of iron. These include: iron sensors based on 1,10-phenanthroline entrapped in sol-gel glass [2,3] ; various fluorescent probes used to detect iron in biological systems [4] ; and ferric ion biosensors using fluorescent siderophores such as azotobactin δ [5], parabactin [6], and pyoverdin [7-10] .

Pyoverdin (also called pyoverdine or pseudobactin) is an extracellular fluorescent siderophore produced by some *Pseudomonas *bacteria growing in low iron environments. There are more than 60 different pyoverdin molecules identified to date, and they all consist of a dihydroxyquinoline chromophore attached to a variable peptide chain (6 to 12 amino acids, of L- and D-form) and a variable side chain [11]. Pyoverdin has potential as an iron biosensor because the fluorescence is quenched by the binding to ferric ion [7].

Previously, pyoverdin from *P. fluorescens *was immobilized on controlled pore glass to produce a biosensor for ferric ion [7] and a biosensor for ferric ion and total inorganic iron [9]. Also, a ferric ion biosensor was developed by immobilizing pyoverdin (from *P. fluorescens*) in sol-gel glass [8]. In these three iron biosensors based on pyoverdin, the interference due to ferrous ion (Fe^2+^) and other metal cations was studied. The fluorescent response of pyoverdin to the Fe^3+ ^concentration changed very little when Fe^2+ ^was added, even at a concentration one hundred times that of Fe^3+^. Thus, it was assumed that pyoverdin was not bound significantly to ferrous ion. However, other research indicates that pyoverdin binds strongly to ferric ion and slightly to ferrous ion [12,13]. Previous work by Xiao and Kisaalita [14] with pyoverdin from *P. fluorescens*, showed that pyoverdin can bind (and oxidize) Fe^2+^. In research with pyoverdin immobilized in mesoporous templated silica, interference due to other metals was studied but ferrous ion was not tested [15]. A ferric ion biosensor using *P. fluorescens *culture solution (containing pyoverdin) showed no significant interference due to Fe^2+ ^[10].

The main objective of this research was to determine the effect of ferrous and ferric ions on the fluorescence of pyoverdin immobilized in porous sol-gel glass. Based on the research of Barrero et al. [8], it was hypothesized that the response of pyoverdin to iron would change when it was immobilized in sol-gel glass. Pyoverdin from *P. aeruginosa *was immobilized in small cylindrical pellets (approx. 3.6 mm height × 3.4 mm diameter) of sol-gel glass. Three formulations of sol-glass were studied because the formula and method used to produce a doped sol-gel glass can influence the reactivity of the immobilized biomolecule [16]. The doped sol-gel glass pellets were characterized using an iron leaching approach and scanning electron microscopy. A flow cell design enabled the measurement of the fluorescence of the pyoverdin-doped pellets in a standard fluorometer. The three formulations of pyoverdin-doped sol-gel glass were tested for iron specificity (if pyoverdin binds more specifically to Fe^2+ ^or Fe^3+^). These results were also compared to the iron specificity of pyoverdin in solution, and further optimization of the iron biosensor was considered.

## Methods

### Microorganism and pyoverdin production

*Pseudomonas aeruginosa *ATCC 15692 was streaked on a slant of nutrient broth yeast extract (NBY) medium. After incubation at 37°C overnight, these cells were used to inoculate precultures of 50 ml of an iron-low synthetic succinate medium, in 125 ml Erlenmeyer flasks. This succinate medium contained, per liter, 7.86 g of K_2_HPO_4 _• 3H_2_O, 3.0 g of KH_2_PO_4 _, 1.0 g of (NH_4_)_2_SO_4 _, 0.1 g of MgSO_4 _• 7H_2_O, and 4.0 g of succinic acid. The pH was adjusted to 7 by adding 1.0 M NaOH, and the medium was sterilized by autoclaving [17]. The preculture was incubated at 37°C for 6-7 hours while being shaken at 200 rpm, in a New Brunswick Innova 4000 incubator-shaker. Aliquots of 10 ml of the preculture were used to inoculate cultures of 200 ml of succinate medium, each in a 500 ml Erlenmeyer flask. These culture flasks were incubated at 37°C for 15-18 hours (until the end of log phase growth) while being shaken at 200 rpm. The cultures were then centrifuged (10,000 g for 10 min, at 4°C), and the supernatant was filtered (0.2 microns). This cell-free solution, termed "crude pyoverdin", was a mixture of pyoverdins that had been produced extracellularly by the cells, along with the salts of the succinate medium.

### Pyoverdin Isolation

Pyoverdin was isolated from the crude solution by copper-chelate chromatography [18], using a Chelating Sepharose Fast Flow column (1.5 × 25.0 cm; Pharmacia LKB Biotechnology). This column had been presaturated with CuSO_4 _and equilibrated with 20 mM HEPES (N-[2-hydroxyethyl]piperazine-N'-[2-ethanesulfonic acid]) buffer (pH 7.0, containing 100 mM NaCl). The crude pyoverdin was lyophilized, dissolved in deionized water, and then applied to the column. The column was first eluted with 200 ml of the 20mM HEPES buffer (pH 7.0), then with about 250 ml of 20 mM acetate buffer (pH 5.0), and finally with 200 ml of 20 mM acetate buffer (pH 4.0). The elution rate was 60 ml/hr. A total of five samples of the lyophilized crude pyoverdin, 1.0 gram each, were fractionated. Between 58 and 80 fractions (5 - 10 ml each) were collected during each fractionation. Each fraction was analyzed by fluorescence (excitation/emission at 400/460 nm) and absorbance (400 nm) spectroscopy to establish five chromatograms showing the peaks.

### Pyoverdin purification

Those fractions making up the largest peak from each chromatogram were pooled together for further purification [18]. This solution was lyophilized, dissolved in 10 mM EDTA (ethylenediaminetetraacetic acid), and purified with a Sephadex G-15 column (1.5 × 100 cm). The column was eluted with ultrapure deionized water, and 45 fractions (4 to 4.5 ml each) were collected. A sample from each fraction was diluted in 0.1 M acetate buffer (pH 5.0) and fluorescence (exc./emis. at 400/455 nm) and absorbance (200-800 nm) were measured. The fraction having the highest fluorescent yield was chosen as the purified pyoverdin to be used in the rest of the experiments. The concentration of pyoverdin in this purified fraction was determined by spectrophotometric titrations with ferric iron, as described in the next section.

At high pH, ferric ion is very insoluble and ferrous ion can readily oxidize to ferric ion. Thus, in this research, all experiments were conducted with pyoverdin in 0.1 M acetate buffer at pH 5.0, similar to other previous studies [13,14,17,19].

### Fluorometric titration of purified pyoverdin

A fluorometric, linear-segment, end point titration [20], based on a procedure described by Chen et al. [13], was followed. As increased amounts of ferric iron are added to a pyoverdin solution, the fluorescence continues to be quenched until an increase in iron no longer results in further fluorescence quenching. At this titration point the iron concentration is equal to the pyoverdin concentration, assuming equimolar binding of iron and pyoverdin [13].

A pyoverdin stock solution was made containing 0.995 μl purified pyoverdin per 2.0 ml 0.1 M acetate buffer, pH 5.0. For each fluorescence titration data point, 2.0 ml of the pyoverdin stock solution was placed in an acrylic cuvette (Sarstedt no. 67.755, 10 × 10 × 48 mm) with a micro stirrer bar. Each cuvette (at room temperature, 25°C) was placed in a Luminescence Spectrometer (Perkin Elmer LS50B) and fluorescence was measured (with constant stirring) at the wavelengths of maximum excitation and emission, 390 and 452 nm, respectively.

An increased amount of iron solution was added to each successive cuvette. The iron solutions were: 5 - 70 μl of 89.53 μM (5 ppm) ferric chloride solution or 15 - 35 μl of 358.12 μM (20 ppm) ferric chloride solution. Since the iron solution was made up in 20 mM HCl, an additional aliquot of 20 mM HCl was added to each cuvette, to maintain a constant total amount of HCl (at 70 μl) for each cuvette.

For each data point (each cuvette), the fluorescence was measured for 5 minutes to obtain a stable initial reading, the required aliquot of 20 mM HCl was added and the fluorescent scan continued for another 5 minutes. Then the iron solution was added and the scan continued for 20 minutes. Additional fluorescence measurements were made at 1 hour and 24 hours after the addition of iron. The fluorometric titration was replicated three times. The data were analyzed by comparing the fluorescent intensity (relative fluorescence units, rfu) at 3 min, 10 min, 20 min, 1 hr, and 24 hr after the ferric solution was added.

### Absorbance titration of purified pyoverdin

To validate the pyoverdin concentration determined fluorometrically, an absorptive end point titration with ferric iron was conducted, again assuming equimolar binding of iron and pyoverdin. Previous research has shown that the absorbance (450-460 nm) of pyoverdin increases when it forms a complex with iron [14,17]. It is assumed that the iron concentration equals the pyoverdin concentration at the point at which further additions of iron to a solution of pyoverdin cause no further increase in absorbance [17].

Absorbance measurements were made with a Beckman DU 650 Spectrophotometer. The titration was conducted with 2.0 ml of a dilute pyoverdin solution, which contained 9.9 μl purified pyoverdin and 1.99 ml 0.1 M acetate buffer, pH 5.0. The initial absorbance (460 nm) of this pyoverdin solution was measured, and then 10 μl of 1.791 mM (100 ppm) ferric chloride was added to the cuvette, and the absorbance was measured at 1 minute intervals for 10 minutes. Another 10 μl of the iron solution was added, and the absorbance was again measured at 1 minute intervals for 10 minutes. This 10 μl addition of iron was repeated a total of 5 times, until 50 μl of the iron solution had been added, resulting in a final iron concentration of 43.67 μM in the cuvette. Two replications of the absorbance titration were conducted.

The absorption spectrum of a solution of purified pyoverdin (in 0.1 M acetate buffer, pH 5.0) was analyzed. From a plot of absorbance (at a specific wavelength) versus molar concentration, the molar absorptivity (ε) was determined from the slope of the line.

### Sol-gel preparation and characterization

A simple change in the formulation of sol-gel glass can change the chemical and physical properties of the final material. One property that can change is the porosity [21,22], which can affect the reaction kinetics of the immobilized biomolecule. In this study, three different formulations (A,B,C) of pyoverdin-doped sol-gel glass were prepared, and the only variable changed between sol-gel formulas was the amount of water added. The three formulations were based on a slight modification of the formula used by Dai et al. [23]. Table 1 lists the components (solutions) for each, along with the calculated R value and the calculated amount of pyoverdin per pellet. Since there was a different amount of water added in each formula, but the amount of pyoverdin remained the same (0.04 ml), the final concentration of pyoverdin in each formulation varied slightly. Tetramethyl orthosilicate (TMOS, Sigma-Aldrich Co., St. Louis, USA) was 98%, methanol (Fisher Scientific) was HPLC grade with 0.01% water, and water was ultrapure, deionized to 18 MΩ-cm (through Barnstead NANOpure^® ^Infinity UF, Barnstead/Thermolyne, Dubuque, Iowa, USA).

**Table 1:** Pyoverdin-doped sol-gel glass formulations

*Sol-Gel formulation*	*A*	*B*	*C*
methanol (ml)	9.6	9.6	9.6
2 N HNO_3 _(ml)	7.0	7.0	7.0
water (ml)	0	3.0	6.0
pyoverdin^1 ^(ml)	0.04	0.04	0.04
TMOS^2 ^(ml)	9.6	9.6	9.6
			
R value^3^	5.62	8.20	10.78
number of pellets made	80	92	100
pyoverdin per pellet^4 ^(μl)	0.50	0.43	0.40

^1 ^purified pyoverdin solution

^2 ^tetramethyl orthosilicate

^3 ^calculated as: (moles water)/(moles silicon)

Water sources - nitric acid, water, and pyoverdin solution.

^4 ^calculated as: (40 μl pyoverdin solution)/(# pellets made)

The solutions were mixed in a 100 ml glass (Pyrex) beaker in the order listed in Table 1. Pellets of the sol-gel glass were produced by pipetting 270 μl of the sol-gel solution into each well of a 96-well micro culture plate (BD Falcon™ #3072, polystyrene, Becton Dickinson and Company, New Jersey, USA). The lid was placed on the plate and sealed with tape for about 2 weeks. The tape was then removed without opening the lid. After 4 more days, the lids were propped open for further drying of the pellets. The resulting air-dried sol-gel glass is called "xerogel". The slow rate of drying prevented cracking of the pellets. All sol-gel procedures and drying were carried out at room temperature (23 - 27°C).

To characterize the resulting sol-gel glass pellets, a leaching experiment and scanning electron microscopy were performed. An iron leaching experiment, similar to that described by Laughlin et al. [24], was replicated three times to determine the relative porosity of the three sol-gel formulas. Three separate batches of sol-gel glass pellets (A,B,C) were prepared as described above, with the following exception: 50 μl of 20 mM ferrous sulfate was added to each instead of 40 μl of purified pyoverdin. After curing for over a month, 20 pellets of each of the 3 formulas were weighed and placed in a 15-ml plastic centrifuge tube and 3.0 ml ultrapure deionized water was added to each tube. The tubes were gently shaken for 30 seconds, 0.5 ml of the solution was removed for iron analysis, and 0.5 ml water was added to each tube. Additional 0.5 ml samples of the iron leachate were removed from each tube (with fresh water replacement) at the following times: 3.5, 13.5, 23.5, 33.5, and 63.5 minutes of soaking in water. For one of the replications of the experiment, leachate samples were also collected at 123.5 and 243.5 minutes.

The 0.5 ml samples of iron leachate were analyzed for iron concentration with a Sigma Diagnostics^® ^Iron and Iron-Binding Capacity assay (Sigma Diagnostics, St. Louis, MO, USA). Their protocol (Procedure No. 565) for total iron was followed. This colorimetric method (absorbance at 560 nm) uses ferrozine. Sigma iron standards were included in each assay.

Scanning electron microscopy (SEM) was used to obtain digital images of the internal structure of the three types of sol-gel glass. Fractured surfaces of the pellets were sputter coated with gold and then imaged with a JSM-5800 Scanning Electron Microscope (JEOL USA, Inc., Peabody, MA). The images were enhanced (for clarity) with Adobe^® ^Photoshop^® ^software. The internal structure of these sol-gel glasses was characterized by measuring the diameter of the particles (spheres) in a 400 nm × 400 nm area in the center of each image.

### Flow cell system

**Figure 1:**
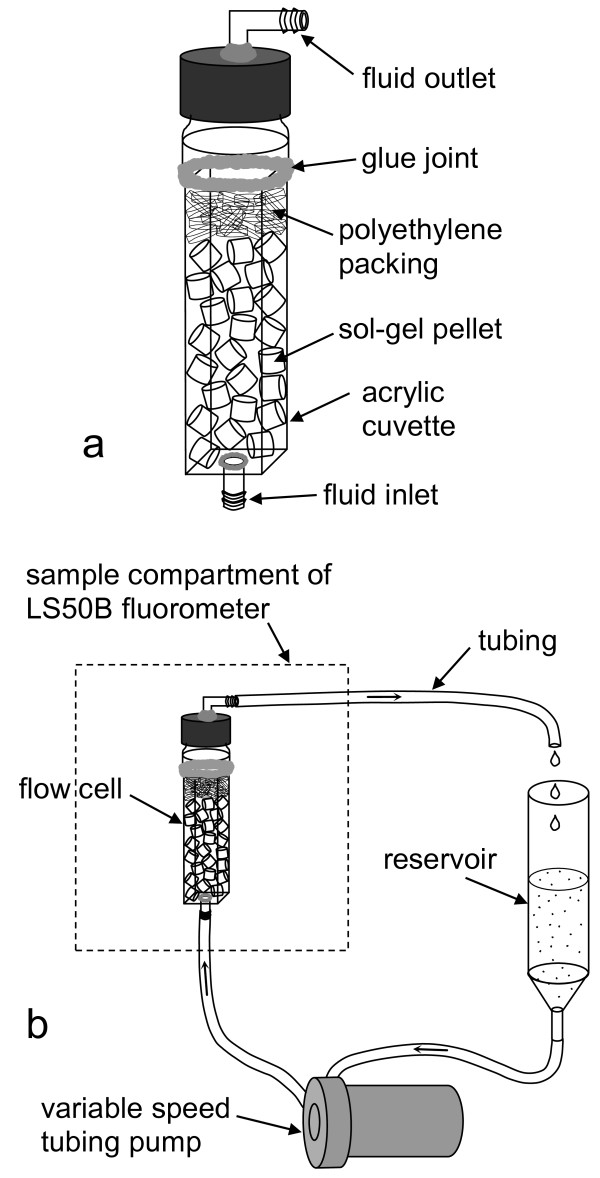
**Flow cell and flow cell system.** a) Flow cell. b) Flow cell system (not to scale).

A flow cell was designed (Figure 1a) which permitted measurement of the fluorescence of pyoverdin-doped sol-gel pellets with a Perkin Elmer LS50B Luminescence Spectrometer (Perkin-Elmer Ltd., Beaconsfield, Buckinghamshire, England). The body of the flow cell was an acrylic cuvette (Sarstedt, no. 67.755, 10 × 10 × 48 mm) with a hole drilled in the bottom. A plastic barbed fitting (3.5 mm I.D.) was glued to this hole to form the inlet. The upper section of the flow cell was the top of a small screw-capped glass vial, which was cut off and glued to the top of the cuvette. A hole was drilled into the screw cap and a plastic barbed fitting was glued to this hole to form the outlet. Sol-gel pellets were held in place with polyethylene ribbon (cut from a disposable transfer pipet) to avoid fluctuations in the fluorescence signal. The flow cell was placed in a single cell cuvette holder (modified by drilling a hole through the bottom) that fits in the sample compartment of the LS50B.

In the flow cell system (Figure 1b), solutions were circulated through the flow cell with a variable speed pump. Solutions were added to the system through a reservoir constructed from a 50 ml plastic centrifuge tube with a plastic hose barb glued to a hole drilled in the end. The reservoir, pump, and flow cell were connected with Tygon^® ^tubing.

### Iron specificity of pyoverdin immobilized in sol-gel glass

To determine the iron specificity of each formulation of pyoverdin-doped sol-gel glass, 22 sol-gel pellets were weighed and placed in the flow cell. Polyethylene ribbon was packed into the top of the flow cell, and the flow cell system (flow cell, tubing, and reservoir) was filled with 35 ml of 0.1 M acetate buffer, pH 5.0. The pump was set for a flow rate of 63 ml/min, to continuously circulate the acetate buffer through the flow cell. Because the spectra of pyoverdin were altered upon immobilization, the fluorescence was measured at an excitation and emission of 382 and 480 nm, respectively. Iron solutions of 500, 750, and 1000 μl of 20 mM ferrous sulfate or 20 mM ferric chloride were added to the flow cell reservoir at specified times, yielding respective final concentrations of 0.282 mM (16 ppm), 0.420 mM (23 ppm), and 0.556 mM (31 ppm) iron in the flow cell system.

Before addition of iron, separate fluorescent intensity determinations were averaged for the first 3-5 minutes to obtain a value for the initial fluorescent intensity. For each quenching reaction, iron solution was added to the reservoir and within seconds the iron was in the flow cell and circulating. The fluorescence continued to be measured for about 45 minutes. To reuse the pyoverdin-doped sol-gel pellets for another quenching reaction, a regeneration procedure, similar to that used by Barrero et al. [8], was followed. Iron bound to immobilized pyoverdin was removed by pouring 1 M HCl into the reservoir while allowing all the acetate buffer to drain out of the flow cell system. The HCl was circulated for 20-30 minutes and then drained as fresh acetate buffer was reintroduced into the system. The acetate buffer was circulated for about an hour, or until the fluorescent intensity had stabilized. This completed the regeneration of the sol-gel pellets and the next quenching reaction was started.

For sol-gel A, the ferric and ferrous quenching experiments were both conducted on the same group of 22 pyoverdin-doped pellets. However, separate groups of 22 pellets were used for the ferric and ferrous quenching experiments with sol-gels B and C. Analysis of the data for sol-gel A indicated that the order in which the effects of ferrous or ferric iron were tested did not affect the results (data not presented).

### Iron specificity of a solution of purified pyoverdin

To determine the iron specificity of a solution of pyoverdin, the procedure described in the section *Fluorometric titration of purified pyoverdin *was followed, except that both ferric and ferrous iron solutions were used. For each data point, 2.0 ml of the pyoverdin stock solution was added to an acrylic cuvette. The iron solutions added were: 10, 30, 40, 50, and 70 μl of 89.53 μM (5 ppm) ferrous sulfate, for final concentrations of 0.45, 1.32, 1.76, 2.18, 3.03 μM ferrous iron, respectively; and 10, 30, 50, and 70 μl of 89.53 μM (5 ppm) ferric chloride, for final concentrations of 0.45, 1.32, 2.18, and 3.03 μM ferric iron, respectively. As in the fluorometric titration, the fluorescence was measured continuously (exc./emis. at 390/452 nm) with stirring, but for a total of 50 minutes. After the first 5 minutes the aliquot of 20 mM HCl was added and after the next 5 minutes the iron solution was added. The data collected for the remaining 40 minutes revealed how the fluorescence was quenched by iron.

## Results

### Pyoverdin isolation and purification

**Figure 2:**
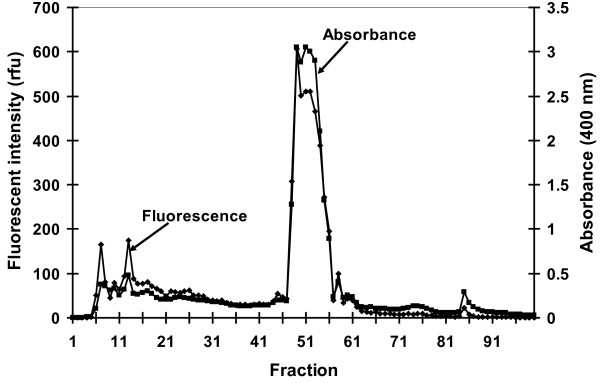
**Copper-chelate chromatogram for sample #5 (*P. aeruginosa *15692), showing the large pyoverdin peak (fractions #48 - #56)**.

The chromatograms of the five samples of lyophilized crude pyoverdin fractionated by copper-chelate chromatography were very similar. Minor variations occurred in the location and size of the peaks, but in each case one very large peak eluted, as shown in the representative chromatogram for sample #5 (Figure 2). Those fractions comprising the large peak for each sample (fractions #48 - #56 for sample #5) were then pooled together and purified in the Sephadex column.

From fluorescence and absorbance measurements on the Sephadex purified fractions (data not shown), fractions #9 - #11 had large fluorescent yields, with fraction #10 the greatest. These 3 fractions had the same maximum excitation and emission wavelengths, and they exhibited rapid quenching by ferric ion. Fraction #10 purified pyoverdin in 0.1 M acetate buffer (pH 5.0) had maximum excitation at twin peaks of 390 and 405 nm and maximum emission at twin peaks of 450 and 460 nm. Thus, for experiments using a solution of pyoverdin, the fluorescence was measured at an excitation and emission of 390 and 452 nm, respectively. In all subsequent experiments referring to purified pyoverdin, fraction #10 was used.

### Spectrophotometric titrations of purified pyoverdin

**Figure 3:**
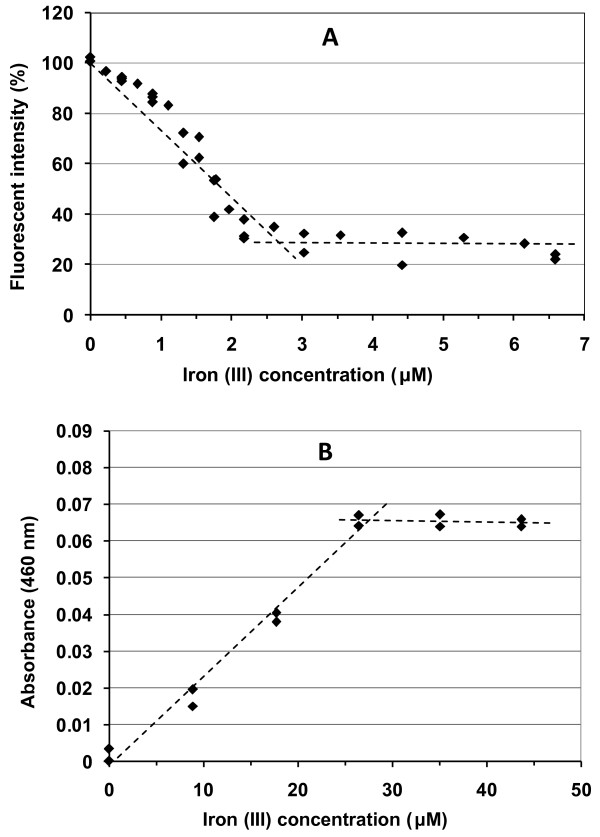
**Fluorescence and absorbance titrations**. A) Fluorescence titration of purified pyoverdin (3 replicates). Data points indicate the fluorescence 10 min. after each iron (III) addition. (ex/em = 390/452 nm) B) Absorbance titration of purified pyoverdin (2 replicates). Data points indicate the absorbance 10 min. after each iron (III) addition.

The fluorometric titrations showed that for all iron concentrations, most of the fluorescence quenching occurred within the first 3 minutes, but nearly 24 hours was required for complete quenching with the higher concentrations of iron. Yoder and Kisaalita [25] show data for the fluorescence quenching of pyoverdin after 24 hours. Fluorescence data (three replications) at 10 minutes after the addition of iron (Figure 3A) are the most representative and are comparable to the other experiments. Some variation in the initial fluorescent intensity (before iron was added) occurred between data points for each replication and between replications. This variation was compensated for by using the percent fluorescent intensity, calculated as: fluorescence (%) = ( F_10_/F_I _) × 100, where F_10 _is the fluorescent intensity (rfu) 10 min. after the addition of iron, and F_I _is the initial fluorescent intensity (rfu) before the iron solution was added.

For fluorometric titration completed in triplicate, at the intersection of two linear segments, the titration point was established at 2.9 μM iron (III) (Figure 3A). Assuming equimolar binding of iron to pyoverdin and accounting for the dilution in the cuvette, the concentration of purified pyoverdin in fraction #10 was calculated at 6.0 mM.

For absorbance titration completed in duplicate, at the intersection of two linear segments, the titration point was established at 28 μM iron (III) (Figure 3B). Accounting for the dilution in the cuvette, the concentration of purified pyoverdin in fraction #10 was calculated at 5.7 mM. As the fluorometric method is considered more sensitive than the absorbance method, a concentration of 6.0 mM purified pyoverdin was assumed.

The absorption spectrum of the purified pyoverdin in 0.1 M acetate buffer (pH 5.0) showed two peaks with maxima at 367 nm and 380 nm. The calculated molar absorptivities were ε = 4440 cm^-1^M^-1 ^at 380 nm, and ε = 0 cm^-1^M^-1 ^at 460 nm. For the ferri-pyoverdin complex in an excess of ferric ion, ε = 3140 cm^-1^M^-1 ^at 460 nm.

### Characterization of sol-gel glass

After drying for several months the sol-gel pellets were approximately cylindrical in shape with a height of about 3.6 mm and diameter of 3.4 mm. The volume of each dried pellet was only 11.9% of the original volume. Measurements made on random samples of 6 pellets taken from each formulation showed a small difference in size (Table 2). Because B and C sol-gel pellets were made with a larger proportion of water, these pellets shrank more and were slightly smaller than A sol-gel pellets. Although there was a variation in the size of pellets and pyoverdin per pellet (Table 2), dividing the pyoverdin per pellet by the average volume of the pellet yields the following values for sol-gels A, B, C, respectively: 0.088, 0.083, 0.078 × 10^-3 ^μmoles pyoverdin per μl pellet volume. In this regard the dried pellets were very similar.

**Table 2:** Pyoverdin-doped sol-gel glass pellets after drying

*Sol-Gel formulation*	*A*	*B*	*C*
pellet dimensions^1^			
height (mm)	3.45 ± 0.48	3.73 ± 0.41	3.65 ± 0.21
diameter (mm)	3.56 ± 0.19	3.27 ± 0.08	3.28 ± 0.17
volume (μl)	33.99 ± 1.57	31.26 ± 3.30	30.87 ± 1.74
			
pyoverdin solution			
per pellet (μl)	0.50	0.43	0.40
			
pyoverdin per pellet^2^			
(μ moles)	0.003	0.00258	0.00240

^1 ^average ± standard deviation;

pellets were short circular cylinders

^2 ^calculated as:

(pyoverdin solution per pellet) × [P],

where [P] = 6.0 × 10^-3 ^μmoles pyoverdin/μl pyoverdin solution

**Figure 4:**
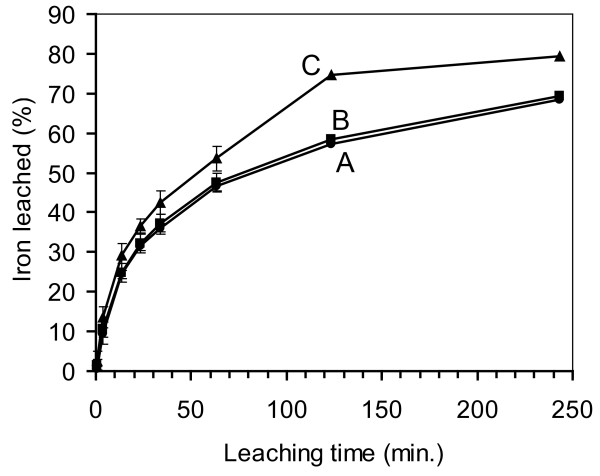
**Leaching of iron (II) from ferrous sulfate-doped sol-gel pellets**. The iron concentration initially in the pellets was set to 100%. Data points are the means of 3 replications. Error bars indicate ± standard deviation.

The results of 3 replications of the iron leaching experiment are presented (Figure 4). The total amount of iron calculated to be in each group of pellets was set to 100%. These iron-doped pellets had cured for about 6 weeks. The data were statistically analyzed using a t-test (SigmaPlot^® ^ver. 3.03, Jandel Corp.) to compare the means at each sampling time. There was significantly (α = 0.05) more iron leached from sol-gel C than from sol-gels A and B at leaching time 63.5 minutes. At times 23.5 and 33.5 minutes, the iron leached from sol-gel C was different from A, but not different from B. These results show that all three formulations of sol-gel glass were quite porous to ferrous ions, but sol-gel C had the greatest porosity.

**Figure 5:**
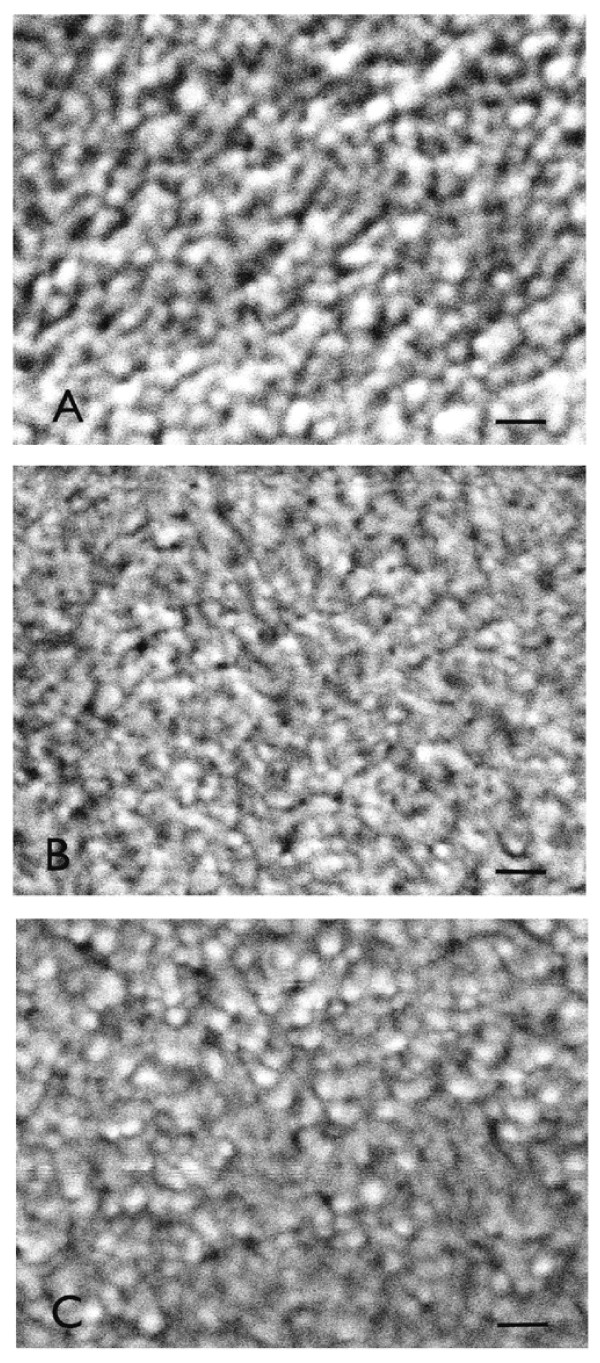
**Scanning electron micrographs of**: A) sol-gel glass A; B) sol-gel glass B; C) sol-gel glass C. The bar in the lower right of each micrograph is 80 nm.

Scanning electron micrographs of sol-gels A, B, and C (Figure 5) revealed a globular morphology for all three formulations. In each sol-gel type there appeared to be a variation in the size of particles. Upon analysis of the images, the average particle diameter (nm) and standard deviation (in parentheses) of the three glasses were: sol-gel A, 25.2 (4.9); sol-gel B, 19.4 (3.0); sol-gel C, 22.8 (4.6). Thus, sol-gel A was composed of slightly larger sol-gel polymer particles. As the pore spaces are interconnected areas between the polymer particles, no attempt was made to measure them.

### Iron specificity of pyoverdin immobilized in sol-gel glass

**Figure 6:**
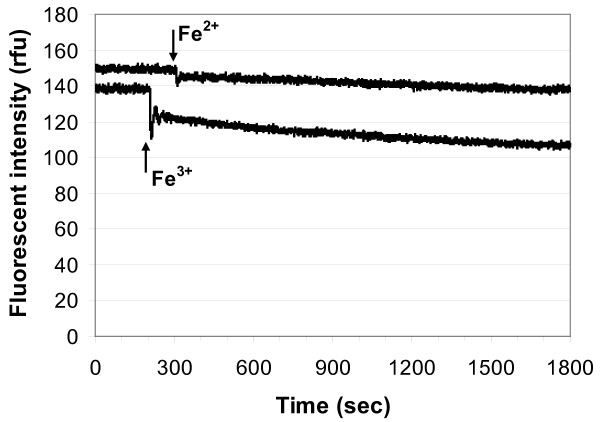
**Fluorescence quenching of pyoverdin immobilized in sol-gel B**. 1.0 ml of either 20 mM ferric chloride (Fe^3+^) or 20 mM ferrous sulfate (Fe^2+^) was added at the times indicated by the arrows, for a final iron concentration (II or III) of 0.555 mM. (ex/em = 382/480 nm)

Pyoverdin immobilized in sol-gel glass responded differently to ferric and ferrous ions. As shown in a representative plot for sol-gel glass B (Figure 6), there was an initial large, fast quenching of the fluorescence due to ferric ion (0.556 mM) followed by a gradual decrease. In response to ferrous ion (0.556 mM) there was an initial fast, but very small decrease in fluorescence followed by a very gradual decrease. Given the noise of the baseline signal, the ferrous response shown (Figure 6) would be close to the detection limit. Similar responses to ferric and ferrous ion were observed in all 3 formulations of pyoverdin-doped sol-gel glass.

**Figure 7:**
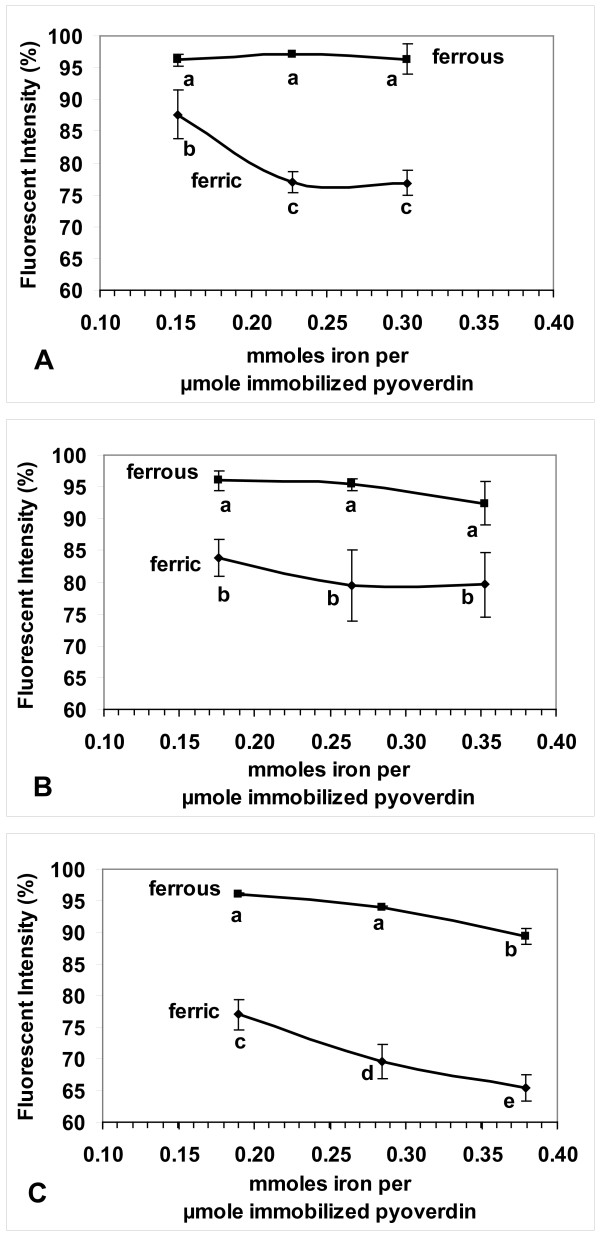
**Fluorescence quenching of pyoverdin immobilized in: A) sol-gel A, B) sol-gel B, C) sol-gel C**. Data points are mean values of fluorescence 10 min. after the addition of iron (II or III). Error bars indicate ± standard deviation. Means labeled with the same letter are not significantly different (α = 0.05). The initial fluorescent intensity (before addition of iron) was set to 100%. (ex/em = 382/480 nm)

For each sol-gel formulation (A,B,C), at each concentration of iron (0.282, 0.420, 0.556 mM), between 2 and 7 replications of the quenching reaction were conducted. Because the data were thus not balanced, they were statistically analyzed using a General Linear Models (GLM) procedure (PC SAS, ver. 8, SAS Institute). The means and standard deviations (shown as error bars) of the fluorescent intensity (%) at 10 minutes after the addition of iron were plotted (Figure 7). In each of the 3 plots, means labeled with the same letter are not significantly different (α = 0.05). To correct for the slightly different amounts of pyoverdin in the groups of pellets, the units for the x-axis are mmoles of iron per μmoles of immobilized pyoverdin. Thus, at any particular ratio of the amount of iron in the flow cell system to the amount of pyoverdin in the flow cell system, the fluorescent responses of the three formulations of pellets can be properly compared. For all 3 sol-gel formulations the fluorescence was quenched significantly more by ferric ion than by ferrous ion. For pyoverdin immobilized in sol-gels A and B, a very small amount of fluorescence quenching was due to ferrous ion, regardless of the iron concentration. In sol-gel C a small but linear change in the quenching was due to ferrous ion. For sol-gels A and C, there were significant (α = 0.05) effects due to the iron concentration, and due to the interaction between iron type (II or III) and iron concentration. Pyoverdin immobilized in sol-gel C had the greatest specificity for the iron type and the greatest sensitivity (and linearity) to the change in iron concentration.

### Iron specificity of a solution of purified pyoverdin

**Figure 8:**
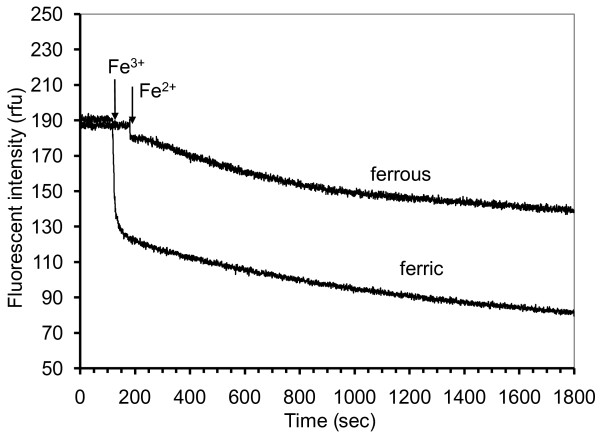
**Fluorescence of purified pyoverdin solution (3.0 μM) in response to 70 μl of either 89.5 μM ferric chloride (Fe**^**3+**^**) or 89.5 μM ferrous sulfate (Fe**^**2+**^**)**. Each iron solution was added at the time indicated by the arrow, for a final iron (II or III) concentration of 3.0 μM. (ex/em = 390/452 nm)

An aqueous solution of purified pyoverdin also responded differently to ferric and ferrous ion. A representative plot of the response of purified pyoverdin in solution (3.0 μM) to either ferric or ferrous ion (3.0 μM) is shown (Figure 8). Ferric ion caused a fast, large initial fluorescence quenching and ferrous ion caused a fast but small initial fluorescence quenching. The fluorescence continued to decrease gradually at the same rate for both ions.

**Figure 9:**
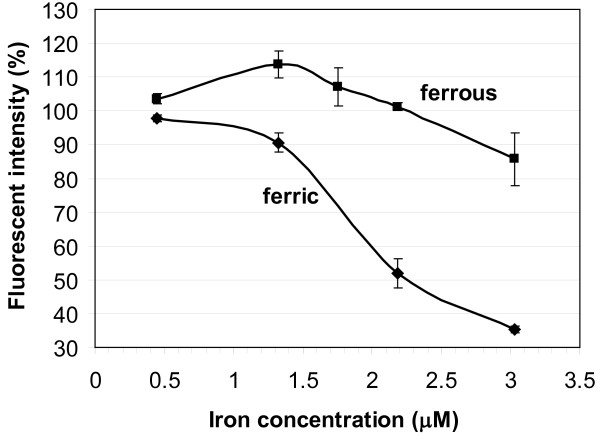
**Fluorescence of purified pyoverdin solution (3.0 μM) in response to ferrous and ferric ion**. Data points are mean values of fluorescence 10 min. after the addition of iron (II or III). Error bars indicate ± standard deviation. The initial fluorescent intensity (before addition of iron) was set to 100%. (ex/em = 390/452 nm)

For each type of iron (II or III), at each concentration, 3 or 4 replications of the quenching reaction were conducted. The means and standard deviations (shown as error bars) of the fluorescent intensity (%) 10 min. after the addition of iron is presented (Figure 9). The data were statistically analyzed using a t-test (SigmaPlot^® ^ver. 3.03, Jandel Corp.) to compare the means. The quenching of fluorescence by ferric ion was significantly (α = 0.05) greater than the quenching by ferrous ion, for the 4 iron concentrations compared. Due to an unknown mechanism, lower concentrations of ferrous ion caused the fluorescent intensity to increase. The fluorescence of pyoverdin in solution (3.0 μM) exposed to 1.32 μM ferrous iron had increased to 113.8% by 10 min. (Figure 9) and after 30 min. (data not shown) the fluorescence had increased to 128.8%.

## Discussion

### Spectrophotometric titrations of purified pyoverdin

The absorption peaks of 367 and 380 nm for the purified pyoverdin in 0.1 M acetate buffer (pH 5.0) were comparable to absorption peaks previously described for pyoverdins from *P. aeruginosa *[12,17,26,27]. However, the calculated molar absorptivities were much lower than those previously reported [26]. For free pyoverdin, at pH 5.0, at 380 nm, ε = 4440 cm^-1^M^-1 ^(calculated) versus ε = 16500 cm^-1^M^-1 ^(reported) and for the ferric-pyoverdin complex, at 460 nm, ε = 3140 cm^-1^M^-1 ^(calculated) versus ε = 6500 cm^-1^M^-1 ^(reported). The reason for these differences is unclear. Consequently, pyoverdin concentrations were determined from fluorescence and absorbance titration results and not from molar absorptivities.

The fluorescence spectra were very similar to those seen with other pyoverdins [15,18]. Also, both the absorbance and fluorescence spectra were very dependent upon the pH and type of buffer (data not shown), as is widely known for pyoverdins.

Although the fluorometric titration (Figure 3A) was not conducted to determine a detection limit for iron, the data show that changes in iron concentration of 0.3 μM and smaller can be readily detected.

### Characterization of sol-gel glass

The complete characterization of a doped sol-gel glass is not simply a function of the porosity because the immobilization of a biomolecule can be a combination of physical entrapment and ionic interactions between the biomolecule and the sol-gel matrix [16,28]. Research has shown that the retention (lack of leaching) of a molecule doped into a sol-gel glass is not simply a function of the size or molecular weight of the molecule. Other properties of the molecule such as the charge, degree of aggregation, surface activity, solubility, and tertiary structure can greatly influence the retention in the sol-gel glass [29]. Thus, the results of the iron leaching experiment and the SEM images can together reveal characteristics of the three sol-gel glasses.

The results of the iron leaching experiment show that sol-gel C had the greatest porosity relative to ferrous ion. Also, of the three sol-gel formulations, C had the greatest R value (10.78). This increase in porosity with an increase in R value is similar to what other research has shown. Blyth et al. [30] believed that a high water content can increase the pore size in a two-step acid-base catalyzed sol-gel glass. Kortesuo et al. [31] reported that an increase in the R value from 6 to 28 increased both the release rate and the total amount of a drug released from acid-catalyzed, tetraethyl orthosilicate (TEOS) sol-gel glass. But when others studied acid-catalyzed TEOS sol-gel glass at a range of R values between 2 to 6, they found that an increase in R value resulted in a decrease in leaching or porosity [32-34]. However, some caution should be exercised when comparing sol-gel glasses made with TEOS and TMOS. Given the same conditions (relative water concentration; acid or base catalyzed) the hydrolysis of TEOS is much slower than the hydrolysis of TMOS [35].

The porosity could not readily be determined from just the SEM images. However, as the spherical particles making up the sol-gel glasses were between 19.4 - 25.2 nm, this is very comparable to a sol-gel glass prepared in a very similar manner (TMOS precursor, R = 4, one-step acid catalyzed) that had particles between 20 - 40 nm and an average pore diameter of 2 nm [22].

### Iron specificity of pyoverdin immobilized in sol-gel glass

There was a significant difference between the fluorescent response to ferrous and ferric ion for all three sol-gel formulations. However, the differences seen between the sol-gel formulations may have been partly due to differences in the age of the pellets. Sol-gel A pellets had cured for 18 weeks, B pellets had cured for 29 weeks, and C pellets had cured for 42 weeks when the quenching reactions were conducted. As sol-gel glass ages the material shrinks, the pore diameters decrease, and the structure and function of entrapped biomolecules can change [16,28].

During the first eight quenching reactions with sol-gels A and B, as the pellets were repeatedly regenerated the fluorescent intensity was not fully restored, but decreased 4 - 20% after each regeneration, consistent with previous work [8]. For sol-gel C, the fluorescent intensity was fully restored after each regeneration.

When pellets of sol-gels A and B were left in 1 M HCl for extended periods of time (over the weekend) the fluorescent intensity increased for some unknown reason. Pyoverdin seems to have much less tendency to degrade than other biomolecules, perhaps because of the presence of D-form amino acids in its structure [19]. Pyoverdin from *P. aeruginosa *contains two D-serine molecules in the peptide chain [27].

Yoder and Kisaalita [36] reported a decrease in leaching with age for pyoverdin immobilized in sol-gel formulations A, B, and C, at short aging periods of 2 - 6 weeks. An additional study (unpublished data) of pyoverdin-doped sol-gel glass pellets that had aged for 27 weeks and soaked for 14 days in acetate buffer (pH 5.0) showed leaching of pyoverdin. Although the most leaching occurred with sol-gel C, even after 14 days of soaking there was still between 24 - 52% of the initial fluorescence in the three sol-gel formulations. Although pyoverdin leaching was not investigated in the present study, some leaching of pyoverdin was assumed to have occurred. A significant amount of pyoverdin remained in the glasses, as evidenced by the good repeatability of the quenching reactions.

### Iron specificity of a solution of purified pyoverdin

The fluorescence increased when pyoverdin in solution was exposed to low concentrations (1.3 μM) of ferrous ion (Figure 9), but this was not observed with the immobilized pyoverdin. Thus, immobilizing pyoverdin has potential for improving iron specificity, especially at commonly found low iron concentrations. It is reasonable to encounter water samples with 0.07 ppm (1.3 μM) iron because the mean level of iron in well water in Georgia is 0.4 ppm (7.16 μM), but 42% of the well water has no detectable iron (≤ 0.005 ppm) [37]. In this study, specificity investigation was limited to Fe^3+ ^and Fe^2+^. The response of pyoverdin to other metal ions commonly found in well water is reported in a separate study [25].

## Conclusions

For both pyoverdin that was immobilized and pyoverdin in solution, quenching by ferric ion was much greater than by ferrous ion. But, the response to ferrous ion was measurable and cannot be ignored. In several studies of the competition between ferric and ferrous ions in fluorescence quenching of pyoverdin, the response to ferrous ion has been reported as negligible [8-10]. However, in developing an iron biosensor one must consider the possibility of encountering samples that contain predominantly ferrous ion.

The iron specificity of pyoverdin changes when pyoverdin is immobilized in sol-gel glass. And, the specific formulation of the sol-gel glass affects the iron specificity of the immobilized pyoverdin. In this research, pyoverdin immobilized in sol-gel C was the most specific in its binding to ferric ion and it showed the most linear response. Pyoverdin immobilized in two of the sol-gels (A and B) showed very little response to ferrous ion, while pyoverdin in sol-gel C showed a small linear response to ferrous concentration. In contrast, when pyoverdin was in solution, low concentrations of ferrous ion caused an increase in fluorescence by an unknown mechanism, whereas higher ferrous ion concentrations caused a linear decrease in fluorescence.

Optimization of the iron biosensor may be achieved by immobilizing pyoverdin in other formulations of sol-gel glass. Pyoverdin immobilized in a sol-gel thin film should respond more rapidly to analytes due to a much faster rate of diffusion, although there are other problems associated with biosensor thin films [38]. Pyoverdin immobilized in a base catalyzed sol-gel material, having a more branched structure and larger pore spaces [35], may encounter different ionic interactions and the diffusion of analyte may be more rapid, but leaching of pyoverdin may also be a serious problem.

## Competing interests

The authors declare that they have no competing interests.

## Authors' contributions

MY contributed to the design of the study, the acquisition and analysis of data, and the writing of the manuscript. WK contributed to the design and coordination of the study and participated in the writing of the manuscript. All authors read and approved the final manuscript.

## References

[B1] Achterberg EP Holland TW Bowie AR Mantoura RFC Worsfold PJ Determination of iron in seawater Anal Chim Acta 2001 442 1 14 10.1016/S0003-2670(01)01091-1

[B2] Zusman R Rottman C Ottolenghi M Avnir D Doped sol-gel glasses as chemical sensors J Non-Cryst Solids 1990 22 107 109

[B3] Lev O Kuyavskaya BI Gigozin I Ottolenghi M Avnir D A high-sensitivity photometric method based on doped sol-gel glass detectors: determination of sub-ppb divalent iron Fresenius J Anal Chem 1992 343 370 372 10.1007/BF00322873

[B4] Espósito BP Breuer W Cabantchik ZI Design and applications of methods for fluorescence detection of iron in biological systems Biochem Soc Trans 2002 30 729 732 12196179 10.1042/bst0300729

[B5] Palanché T Marmolle F Abdallah MA Shanzer A Albrecht-Gary AM Fluorescent siderophore-based chemosensors: iron (III) quantitative determinations J Biol Inorg Chem 1999 4 188 198 10.1007/s007750050304 10499091

[B6] Lam CKSCC Jickells TD Richardson DJ Russell DA Fluorescence-based siderophore biosensor for the determination of bioavailable iron in oceanic waters Anal Chem 2006 78 5040 5045 10.1021/ac060223t 16841927

[B7] Barrero JM Moreno-Bondi MC Pérez-Conde MC Cámara C A biosensor for ferric ion Talanta 1993 40 1619 1623 10.1016/0039-9140(93)80075-3 18965830

[B8] Barrero JM Cámara C Pérez-Conde MC San José C Fernández L Pyoverdin-doped sol-gel glass for the spectrofluorimetric determination of iron (III) Analyst 1995 120 431 435 10.1039/an9952000431

[B9] Pulido-Tofiño P Barrero-Moreno JM Pérez-Conde MC A flow-through fluorescent sensor to determine Fe(III) and total inorganic iron Talanta 2000 51 537 545 10.1016/S0039-9140(99)00308-2 18967885

[B10] Gupta V Saharan K Kumar L Gupta R Sahai V Mittal A Spectrophotometric ferric ion biosensor from *Pseudomonas fluorescens *culture Biotechnol Bioeng 2008 100 284 296 10.1002/bit.21754 18080345

[B11] Budzikiewicz H Siderophores of the Pseudomonadaceae *sensu stricto *(fluorescent and non-fluorescent *Pseudomonas *spp.) Fortschr Chem Org Naturst 2004 87 81 237 15079896 10.1007/978-3-7091-0581-8_2

[B12] Albrecht-Gary AM Blanc S Rochel N Ocaktan AZ Abdallah MA Bacterial iron transport: Coordination properties of pyoverdin PaA, a peptidic siderophore of *Pseudomonas aeruginosa* Inorg Chem 1994 33 6391 6402 10.1021/ic00104a059

[B13] Chen Y Jurkevitch E Bar-Ness E Hadar Y Stability constants of pseudobactin complexes with transition metals Soil Sci Soc Am J 1994 58 390 396 10.2136/sssaj1994.03615995005800020021x

[B14] Xiao R Kisaalita WS Fluorescent pseudomonad pyoverdines bind and oxidize ferrous ion Appl Environ Microbiol 1998 64 1472 1476 9575133 10.1128/aem.64.4.1472-1476.1998 PMC106172

[B15] Mureseanu M Renard G Galarneau A Lerner DA A demonstration model for a selective and recyclable uptake of metals from water: Fe(III) ions complexation and release by a supported natural fluorescent chelator Talanta 2003 60 515 522 10.1016/S0039-9140(03)00103-6 18969073

[B16] Jin W Brennan JD Properties and applications of proteins encapsulated within sol-gel derived materials Anal Chim Acta 2002 461 1 36 10.1016/S0003-2670(02)00229-5

[B17] Meyer JM Abdallah MA The fluorescent pigment of *Pseudomonas fluorescens*: biosynthesis, purification and physicochemical properties J Gen Microbiol 1978 107 319 328

[B18] Xiao R Kisaalita WS Purification of pyoverdines of *Pseudomonas fluorescens *2-79 by copper-chelate chromatography Appl Environ Microbiol 1995 61 3769 3774 16535157 10.1128/aem.61.11.3769-3774.1995 PMC1388593

[B19] Teintze M Hossain MB Barnes CL Leong J van der Helm D Structure of ferric pseudobactin, a siderophore from a plant growth promoting *Pseudomonas* Biochemistry-US 1981 20 6446 6457 10.1021/bi00525a025 7306518

[B20] Skoog DA West DM Holler FJ Fundamentals of Analytical Chemistry 1992 6 Fort Worth: Saunders College Publishing

[B21] Klotz M Ayral A Guizard C Cot L Tailoring of the porosity in sol-gel derived silica thin layers Bull Korean Chem Soc 1999 20 879 884

[B22] Bryans TR Brawner VL Quitevis EL Microstructure and porosity of silica xerogel monoliths prepared by the fast sol-gel method J Sol-Gel Sci Technol 2000 17 211 217 10.1023/A:1008711921746

[B23] Dai S Shin YS Toth LM Barnes CE Spectroscopic probing of adsorption of uranyl to uranyl-imprinted silica sol-gel glass via steady-state and time-resolved fluorescence measurement J Phys Chem B 1997 101 5521 5524 10.1021/jp970713c

[B24] Laughlin JB Sarquis JL Jones VM Cox JA Using sol-gel chemistry to synthesize a material with properties suited for chemical sensing J Chem Educ 2000 77 77 79 10.1021/ed077p77

[B25] Yoder MF Kisaalita WS Fluorescence of pyoverdin in response to iron and other common well water metals J Env Sci Health Part A 2006 41 369 380 10.1080/10934520500423501 16484070

[B26] Demange P Wendenbaum S Bateman A Dell A Abdallah MA Winkelmann G, van der Helm D, Neilands JB Bacterial siderophores: structure and physicochemical properties of pyoverdins and related compounds Iron transport in microbes, plants and animals 1987 Weinheim: VCH Verlagsgesellschaft 167 187

[B27] Demange P Wendenbaum S Linget C Mertz C Cung MT Dell A Abdallah MA Bacterial siderophores: structure and NMR assignment of pyoverdins Pa, siderophores of *Pseudomonas aeruginosa *ATCC 15692 Biol Met 1990 3 155 170 10.1007/BF01140574

[B28] Gill I Bio-doped nanocomposite polymers: sol-gel bioencapsulates Chem Mater 2001 13 3404 3421 10.1021/cm0102483

[B29] Skrdla PJ Saavedra SS Armstrong NR Reduction of indicator leaching from doped sol-gels by attachment of macromolecular carriers Appl Spectrosc 1999 53 785 791 10.1366/0003702991947577

[B30] Blyth DJ Poynter SJ Russell DA Calcium biosensing with a sol-gel immobilized photoprotein Analyst 1996 121 1975 1978 10.1039/an9962101975 9008410

[B31] Kortesuo P Ahola M Kangas M Yli-Urpo A Kiesvaara J Marvola M In vitro release of dexmedetomidine from silica xerogel monoliths: effect of sol-gel synthesis parameters Int J Pharm 2001 221 107 114 10.1016/S0378-5173(01)00656-1 11397572

[B32] Butler TM MacCraith BD McDonagh C Leaching in sol-gel derived silica films for optical pH sensing J Non-Cryst Solids 1998 224 249 258 10.1016/S0022-3093(97)00481-X

[B33] McDonagh C Bowe P Mongey K MacCraith BD Characterisation of porosity and sensor response times of sol-gel-derived thin films for oxygen sensor applications J Non-Cryst Solids 2002 306 138 148 10.1016/S0022-3093(02)01154-7

[B34] Noire MH Bouzon C Couston L Gontier J Marty P Pouyat D Optical sensing of high acidity using a sol-gel entrapped indicator Sens Actuators B-Chem 1998 51 214 219 10.1016/S0925-4005(98)00193-2

[B35] Brinker CJ Scherer GW Sol-gel Science: The Physics and Chemistry of Sol-gel Processing 1990 San Diego: Academic Press

[B36] Yoder MF Kisaalita WS Leaching behavior of a fluorescent pyoverdin immobilized in sol-gel glass Open Biotech J 2008 2 157 166 10.2174/1874070700802010157

[B37] Bush PB Berisford YC Hitchcock RN Perkins RG Segars WI Tyson AW 1994 Summary of Well Water Testing in Georgia 1995 Cooperative Extension Service, College of Ag. and Env. Sciences, Univ. of Georgia, Athens, GA

[B38] Gupta R Chaudhury NK Entrapment of biomolecules in sol-gel matrix for applications in biosensors: problems and future aspects Biosens Bioelectron 2007 22 2387 2399 10.1016/j.bios.2006.12.025 17291744

